# Uganda's Anti‐Homosexuality Act undermines public health

**DOI:** 10.1002/jia2.26259

**Published:** 2024-05-07

**Authors:** Andrew Mujugira, Timothy Muwonge, Brian Aliganyira, Stephen Okoboi

**Affiliations:** ^1^ The Infectious Diseases Institute Makerere University Kampala Uganda; ^2^ Ark Wellness Hub Kampala Uganda

1

“When elephants fight, it is the grass that suffers”—African proverb

The Uganda Anti‐Homosexuality Act 2023 (AHA) has had a detrimental impact on vulnerable Ugandans. Since its enactment, the AHA has created disincentives for members of key populations (KP) to access testing, treatment and prevention services, negatively impacting Uganda's hard‐earned reputation for excellence in HIV service delivery ^[^ ^1^ ^]^. Building trust with the global HIV community has been a gradual process; once lost, it can be difficult to regain. The repercussions of the AHA on local KP and external healthcare funding will be challenging to rectify.

Numerous studies have shown that laws criminalizing KP harm public health ^[^ ^2^, ^3^ ^]^, regardless of political, personal or other beliefs, by reducing access to crucial HIV services—precisely the opposite of what is needed, given the disproportionately high HIV burden among KP ^[^ ^4^ ^]^. Evidence from 10 sub‐Saharan African countries indicates that countries that criminalized same‐sex behaviours had five times higher HIV prevalence compared to non‐criminalized settings ^[^ ^5^ ^]^. Additionally, countries with recent prosecutions for same‐sex behaviour had a 12‐fold higher HIV prevalence than those without such laws ^[^ ^5^ ^]^. Data from 194 countries revealed that in countries where same‐sex sexual acts were criminalized, there was an 11% decrease in the proportion of people with HIV aware of their status and an 8% decline in viral suppression ^[^ ^6^ ^]^. Therefore, the AHA could undo the progress made in controlling Uganda's HIV epidemic. For example, enthusiasm for conducting research with three of the five KP identified as being at increased risk of HIV acquisition (i.e. men who have sex with men, transgender people and prisoners) has diminished because of concerns for the safety of investigators and research volunteers ^[^ ^7^ ^]^. The effective exclusion of these KP groups from HIV research will hinder the attainment of national 95:95:95 targets ^[^ ^8^ ^]^.

The AHA has already had detrimental effects on KP service delivery, resulting in individuals going into hiding or leaving the country for fear of violence and legal repercussions ^[^ ^9^ ^]^. AHA‐related societal stigma and discrimination, coupled with limited employment opportunities, have led some members of KP communities to sell sex for survival. These circumstances, coupled with decreased access to healthcare, create an ideal environment for HIV transmission—the exact opposite of what has been accomplished through international cooperation to control the HIV epidemic ^[^ ^9^ ^]^. According to anecdotal reports, donor funding for civil society organizations providing KP services has decreased because of concerns over the reduced feasibility of service delivery in the context of the AHA. This is particularly notable given that development partners contribute 42% of Uganda's total health expenditure, compared to only 15% from the government ^[^ ^10^ ^]^.

Uganda is the second largest beneficiary of research funding from the United States National Institutes of Health (NIH) in Africa ^[^ ^11^ ^]^. In 2022, Ugandan institutions received 71% of their total research funding from the NIH ^[^ ^12^ ^]^. Along with other funders like the European and Developing Countries Clinical Trials Partnership (13%) and the Wellcome Trust (10%), the NIH promotes equitable and inclusive research practices involving KP in Uganda. We are grateful for the valuable contributions made by organizations like the NIH and the U.S. President's Emergency Plan for AIDS Relief towards fostering HIV prevention and care in Uganda. As researchers committed to ending the HIV pandemic, we acknowledge that the AHA may lead to the disruption or withdrawal of research or programme funding from these donors ^[^ ^13^ ^]^. However, such actions would harm vulnerable communities (“the grass” in the proverb) that already face countless threats to their safety and wellbeing.

Implementing the AHA has raised challenges in maintaining international ethical norms for research. In August 2023, the Uganda Ministry of Health released a press statement mandating that health services be accessible to all without discrimination. However, in October 2023, the Uganda National Council for Science and Technology, which oversees all research in the country, issued a circular stating that confidentiality in research may be waived to report any criminal offences to the authorities. These conflicting stances from two government entities made it challenging for physician‐scientists to “thread the needle.” They must uphold their Hippocratic Oath to “first, do no harm” but could breach this oath if required to report KP under their care to the authorities.

In April 2024, the Ugandan Constitutional Court declared section 14 of the AHA (which required reporting of acts of homosexuality) unconstitutional. However, by this time, the repercussions of this provision had already been felt. The ethical conduct of research with KP must always include Respect for Persons, Beneficence and Justice, even in the era of the AHA ^[^ ^14^ ^]^. Respect for Persons entails safeguarding the confidentiality of personal information shared by research participants. Beneficence requires researchers to prioritize maximizing benefits while minimizing harms. Justice requires equitable distribution of the benefits of research ^[^ ^14^ ^]^. Furthermore, the Declaration of Helsinki emphasizes the importance of ensuring equitable access to medical research for underrepresented groups, including KP ^[^ ^15^ ^]^. These firmly established ethical principles must always guide research.

We believe Ugandan researchers and regulators can navigate this challenging landscape while protecting research volunteers’ rights and welfare. Ugandan researchers have gained valuable experience through providing safe and inclusive care during previous epidemics, pandemics and civil wars. This has been achieved despite operating within an underfunded and overburdened healthcare system. We acknowledge the resilience of Ugandan KP communities, which are currently facing life‐threatening circumstances. Therefore, we urge all parties involved to focus on public health needs and ensure that healthcare and research remain exempt from politics. As other African countries adopt punitive laws, efforts must be focused on implementing evidence‐based approaches for HIV prevention and treatment rather than repeating past mistakes. As George Santayana famously said, “Those who cannot remember the past are condemned to repeat it.”

We remain dedicated to upholding ethical research practices and safeguarding the rights and welfare of our vulnerable populations. Working collectively with our communities, regulators and development partners, we are committed to pursuing a more equitable future for all individuals impacted by HIV. After all, elephants need grass to survive.

## COMPETING INTERESTS

AM is supported by research funding from the United States National Institutes of Health (grants R01MH130208 and R01TW12672).

## AUTHORS’ CONTRIBUTIONS

AM and TM conceptualized the article. AM wrote the first draft. All authors carefully reviewed and approved the final version of the manuscript.
